# Prognostic Relevance of Circulating 25OHD Fractions for Early Recovery and Survival in Patients with Hip Fracture †

**DOI:** 10.3390/jcm7080193

**Published:** 2018-08-01

**Authors:** Erzsébet Toldy, Antal Salamon, Bernadette Kálmán, Katalin Ágota, Dániel Horváth, Zoltán Lőcsei

**Affiliations:** 1 Institute of Diagnostics, Faculty of Health Science, Szombathely Campus, University of Pecs, 14 Jókai Street, H-9700 Szombathely, Hungary; 2 Department of Traumatology, Markusovszky University Teaching Hospital, 5 Markusovszky Street, H-9700 Szombathely, Hungary; salamon.antal@chello.hu; 3 School of Medicine, Institutes of Laboratory Medicine and Neurology, University of Pecs, 13 Ifjuság Street-floor 7, H-7624 Pécs, Hungary; kalman.bernadette@markusovszky.hu; 4 Molecular Pathology, Markusovszky University Teaching Hospital, 5 Markusovszky Street, H-9700 Szombathely, Hungary; 5 Department of General Internal Medicine, Markusovszky University Teaching Hospital, 5 Markusovszky Street, H-9700 Szombathely, Hungary; agotakata@yahoo.com (K.Á.); locsei.zoltan@markusovszky.hu (Z.L.); 6 Department of Radiology, Markusovszky University Teaching Hospital, 5 Markusovszky Street, H-9700 Szombathely, Hungary; daneel33@gmail.com

**Keywords:** total-25OHD, bioavailable-25OHD, free-25OHD, cut off values, hip fracture, comorbidities, healing, survival

## Abstract

The relation between vitamin-D (VD) status and healing after hip fracture had not been sufficiently addressed. Currently serum total 25-hydroxy-VD (t-25OHD) is the most widely used indicator of VD status. It is unclear whether free or bioavailable VD are better markers of 25OHD availability for tissues. Validity of overall cut-off values of t-25OHD is limited. Objectives: (1) Assess serum levels of circulating forms of 25OHD in patients with hip fracture (PwHF: *N* = 199) compared to active controls without history of fracture (*N* = 102); (2) determine relationship between 25OHD fractions and functional performance after surgery (FPAS) and survival. The t-25OHD; VD binding protein and albumin levels were measured. Comorbidities; lifestyle; FPAS and survival were recorded at seven months. VD deficiency occurred more frequently in PwHF than in controls (72% vs. 38%). Patients with better FPAS showed higher 25OHD in all fractions than with poor FPAS. Controlled by lifestyle; 25OHD levels were independent predictive factors (*p* < 0.001). Good FPAS values forecasted longer survival (OR: 6.5_CI:3.2–13.3_; *p* < 0.0001). All 25OHD forms showed a tendency to predict survival. Mortality rate decreased to 8% in individuals with t-25OHD levels of >22.6–39.5 nmol/L and increased to 14% with >40 nmol/L. These observations highlight the importance of serum 25OHD assessment and moderate VD substitution for healing and survival.

## 1. Introduction

Osteoporosis has become a worldwide public health problem. Vitamin D (VD) deficiency, loss of bone density and demineralization are well known in the elderly [1,2,3]. Low VD levels are associated with impaired Ca resorption and bone loss. With the aging populations, the number of osteoporotic hip fractures is growing worldwide, and is associated with significant mortality [4,5,6,7]. Lee et al. [8] observed that the five-year relative survival after osteoporotic hip fracture was below that of the general population and was comparable with the survival of some cancers.

Functional recovery measures after surgery for femoral neck fractures in the elderly vary and depend on the ability or motivation to participate in rehabilitation programs. According to Folman et al. [9], only 16.7% of institutionalized elderly patients with femoral neck fractures regained their overall functional ability after surgical treatment, and only in 12.9% of them returned to their pre-injury, ambulatory status. Sufficient VD status plays important roles in both fracture prevention and post-surgical recovery, because it improves muscle strength and improves quality of life during healing, while also reducing the risks for chronic diseases such as diabetes, cancer, osteoporosis and cardiovascular events [10,11,12,13]. Nevertheless, the relation between VD status and healing after hip fractures had not been sufficiently addressed [14,15,16,17].

Clinicians are generally familiar with the recommended serum total 25-hydroxy-VD (t-25OHD) concentrations as the most reliable marker of the vitamin D status. However, despite the widespread acceptance of this biomarker, some studies suggested that the t-25OHD measures alone may not properly reflect the relationship between VD supply and certain health outcomes [18,19,20]. Approximately 90–95% of the circulating t-25OHD is tightly bound to a specific binding protein (DBP). The amount of free fraction (f-25OHD) is below 0.1% of the total concentration. Bioavailable 25OHD (b-25OHD) is the sum of f-25OHD and 25OHD loosely bound to albumin, and it is also a very small fraction of the total concentration (~10%). Presently, it is unclear whether free or bioavailable 25OHD may better reflect the availability of 25OHD for tissues [18,19,20,21,22]. However, altogether sufficient data support that f-25OHD or b-25OHD are more biologically relevant than t-25OHD estimates. Powe et al. showed that bioavailable 25OHD (not bound to DBP) highly correlated with bone density, while t-25OHD did not [13]. Therefore, we asked as to which fraction of circulating 25OHD is a better marker for vitamin D status in patients with hip fracture compared to age matched controls without fracture history. Our first aim was to investigate the three 25OHD fractions in these patients as compared to those in the control group, (cross-sectional analyses). The second aim was to determine the preoperative 25OHD levels (t-25OHD, b-25OHD, f-25OHD) and concentrations of the inter-related intact parathyroid hormone (PTHi) and calcium (Ca) levels in patients with hip fractures, and to determine the correlations between patients’ outcomes and levels of different 25OHD fractions. We investigated longitudinally the relationship between the three 25OHD fractions and favorable outcome/survival after hip fracture, controlling for lifestyle, general clinical conditions and the presence of major chronic diseases, all with an effect on VD metabolism. Our hypotheses included that:

(1) The proper vitamin D supply has a good influence on post-operative rehabilitation and survival in patients with hip-fractures.
(2) The need for vitamin 25OHD for the elderly may be less than that recommended by professional guidelines, since higher than 75 nmol 25OHD seldom can be measured in the sera of healthy elderly individuals with active lifestyle.
(3) The free and bioavailable 25OHD fraction calculated with the binding proteins show a better correlation with the parathormone as t-25OHD.

## 2. Material and Methods

This prospective study was carried out between 13 February and 30 September 2013. For each patient, survival was determined in April 2014 by calculating the elapsed time between the date of surgery and the date of recording.

### 2.1. Patient Groups

Altogether 203 patients with first-ever hip fracture were included at the beginning of the study, four of them with pathological fractures were excluded from further investigations. The main characteristics of the remaining 199 patients sorted by sexes are summarized in Table 1. Females were significantly older than males, but the numbers of cases who were above the expected average age in our county [72 years for men (*N* = 38) and 79 years for women (*N* = 91) according to the Hungarian Central Statistical Office in 2013] were not significantly (*p* = 0.088) different between sexes. The patients were hospitalized mainly (*N* = 165; 83%) in sunny seasons (250–300 sunny h/month).

We collected information about the patients’ lifestyle, physical activity and exposure to sunlight, in addition to VD intake and other medical treatment. Other chronic diseases (diabetes mellitus, hypertension, cardiovascular, neurological or psychiatric diseases) were also recorded. Only 13% (*N* = 25) of patients took no medication and had no chronic diseases. 

The major chronic diseases *(MCD)* that influence the metabolism of VD (kidney and liver diseases and malignancies) were also recorded.

A questionnaire was used in which all patients or, if necessary, a designated family member or guardian, provided information regarding lifestyle and living arrangements: daily physical activity, ability to support himself/herself and vitamin substitution, time spent outdoors, chronic diseases, and habit of taking medicaments. 

The data of patients were collected and recorded at the time of hospitalisation during interviews by the treating surgeons and were verified from medical records by internists. Based on lifestyle, patients were divided into two groups: “active lifestyle”—group capable of doing frequent outdoor activity and general self-care and “inactive lifestyle” group—incapable of supporting themselves, spending most of their time indoors and partially or completely dependent on others for daily living. 

All patients were surgically treated in 24 h, 58 with screw fixation and 123 patients with gamma nail fixation. Eighteen cases were performed with prosthetic replacement.

Moreover, patients were divided into two additional categories according to their functional performance 5–10 days after the surgery, before their discharge to home or transmission to a rehabilitation department. The classification was based on records by physicians or physiotherapists. The patient’s activity levels were determined based on the “NORTON scale” and recorded by the nurses in the nursing diary after surgery and before discharge. 

Patient mobilization was assisted by a physiotherapist after surgery according to the patient’s ability. Mobilization was performed from the first postsurgical day to discharge. Each day, the physiotherapist measured and documented mobilization levels. Based on these scores, we could categorized patients into two groups: “good functional performance” was stated when the patient had good cooperation, early mobilization with the assistance of physiotherapist, could be seated outside, and was ambulatory with a walking frame and finally was able to climb the stairs with aid (crutches) one day before discharge. Those patients, who had poor cooperation for early mobilization and were lying in bed without getting up and lacking cooperation with physiotherapeutic aids and nurses were categorized in the “poor functional performance” group. All these data were checked by the surgeon before the discharge of patients. Mortality data of patients were obtained from hospital records at 7–14 months post-surgery (Table 1).

### 2.2. Control Group

Furthermore, 102 likewise elderly (73 ± 10 years 47 men, 55 women) persons with active lifestyles, normal kidney and liver function, without any known malignancy and any fracture in their medical history, served as controls. They were mainly healthy volunteers, including 10 older tennis (hobby) players and their active relatives as well as elderly people who were considered healthy with normal kidney and liver function and normal other laboratory findings. Their study samples were left over blood samples drawn by family doctors during annual health status check-ups. These control individuals lived in the same geographic location and had the same ethnic distribution as the patients with hip fracture.

### 2.3. Sample Collection and Biochemical Methods

Blood samples were drawn at admission, before surgery. The sera were analyzed for routine chemical parameters, including Ca, albumin and for DBP, t-25OHD, PTHi. All measurements were carried out in a fully automated manner in different runs. t-25OHD was measured by a competitive electro-chemiluminescence (ECL) protein binding assay—inter-assay coefficients of variance (CV%): 6.2–9.7%, at decreasing concentrations in the range of 50.0–16.7 nmol/L and limit of detection: 9.8 nmol/L. The PTHi was assessed by a sandwich principle using ECL techniques—inter-assay CV% were 2.6–6.5%, at the dropping concentration range of 676–27 pg/mL (71.5–2.9 pmol/L). The functional sensitivity: 6 pg/mL (0.64 pmol/L) with the same system as t-25OHD was measured (Cobas e411 analyser, Roche, Rotkreuz, Switzerland). Estimations of the DBP levels were carried out by an immunoturbidometric assay using polyclonal rabbit anti human Gc-globulin antibody (A0021, Dako, Glostrup, Denmark), configured for the Modular System (Roche, Mannheim, Germany). The measuring range of DBP method was 25–495 mg/L, the detection limit was estimated to be 7.6 mg/L. The total CVs were 3.5–6.1% at 405–95 mg/L concentrations. Albumin and Ca levels were determined by a colorimetric assay with endpoint methods using Modular analyser (Roche). The inter-assay CV% exceeded 2% in none of the assays.

### 2.4. Evaluations of the Laboratory Findings

We used reference cut-off values given by the manufacturer for estimating DBP (≥244 mg/L), albumin (≥39 g/L) and Ca (2.15–2.55 mmol/L). We also calculated the albumin adjusted Ca concentration (CaAlb). The bioavailable (b-25OHD) and free (f-25OHD) concentrations were calculated after Vermeulen and Bikle [23,24]. 

The VD status was determined by assessing t-25OHD concentrations using cut off values according to the last recommendations [25]: deficiency as <50 nmol/L, insufficiency as 50–75 nmol/L, and optimal supply as >75 nmol/L. Beyond that, we also defined severe deficiency of VD as <20 nmol/L. In addition, we calculated our own cut-off values from the 5th percentile values of the control group (t-25OHD: ≥22.6 nmol/L; ≥b-25OHD: ≥1.9 nmol/L; f-25OHD: ≥4.5 pmol/L; DBP: 244 mg/L; albumin: 31 g/L) and from the 95th percentile value in case of PTHi: 73 pg/mL.

The rate of mortality was analysed on the basis of our own cut-off values at the <5th; 5th–25th; >25th percentiles.

### 2.5. Statistical Methods

The majority of variables were not normally distributed, therefore we used non- parametric statistical models. The results were expressed as percentages for categorical variables and as medians and 25th–75th percentile (Q25–Q75). In case of normal distributions (age, albumin, DBP, Ca), results were given in mean ± standard deviation (SD). We performed Mann-Whitney U, Chi-square test and Kruskal-Wallis ANOVA and median test, Spearman’s rank correlations. Twenty-eight cases with known chronic renal failure (Estimated Glomerular Filtration Rate <60 mL/min/m^2^) were excluded from the analyses of association between PTHi and 25OHD levels. 

We used one-step binary logistic regression to investigate the associations between early physical performance, as a dependent variable and the different inherent (as independent) determinant factors (MCD, sex, age, lifestyle), put into the regression model one by one. The influence of three 25OHD forms and related biomarkers on early functional outcome was assessed by multivariate binary logistic regression adjusted by significant confounder factors (sex, lifestyle). A similar procedure was used to define factors influencing survival (age, kidney/malignant diseases, functional performance after surgery). The effect of biochemical markers (three 25OHD, PTHi, DBP, albumin levels) on survival was assessed by Cox regression adjusting for confounder factors that proved to be significant during the binary logistic regression. Results were expressed as adjusted odds ratios (OR) with the corresponding 95% confidence intervals (CI). A probability level of <0.05 was accepted as significant. The statistical analyses were carried out using SPSS for Windows, version 24 (SPSS, Inc., Chicago, IL, USA).

The studies have been approved by the institutional research ethics committee and have been performed in accordance with the ethical standards as laid down in the 1964 Declaration of Helsinki and its later amendments or comparable ethical standards. Informed consent was obtained from all individual participants included in the study.

## 3. Results

### 3.1. Comparison of the Groups with and without Fracture

VD deficiency occurred almost twice more frequently in the patient group with hip fracture than in the control group (72% vs. 38%) (*p* < 0.001) on the basis of guideline threshold values of t-25OHD. Serious deficiency occurred almost eight times more frequently in the hip fracture group than in the control group (30.2% vs. 3.9%).

The occurrence of VD deficiency in patients with hip fracture—on the basis of own controls’ cut-off values—varied between 29–35% in the three 25OHD fractions. The lowest incidence of deficiency (29%) was observed for f-25OHD and the highest for the bioavailable and total 25OHD values (b-25OHD: 35%; t-25OHD: 34% vs. 5% in the control group).

Table 2 shows the levels of all measured biochemical markers and the calculated b-25OHD and f-25OHD concentrations in patients with hip fracture compared to the control group. 

Significantly lower concentrations of all 25OHD fractions and binding proteins (DBP, albumin) as well as Ca, but not albumin corrected Ca, were observed in patients with hip fracture as compared to the control group. In contrast, significantly elevated PTHi levels were found in the hip fracture patient group when compared to those in the control group. This difference was still present when the patients with renal failure (*N* = 28) were omitted. Secondary hyperparathyroidism occurred (*p* < 0.001) more frequently in patients with hip fracture than in the control group (33% vs. 12%). 

Similarly, significant negative correlation coefficients (−0.42 to −0.47) were observed between the PTHi and the three 25OHD fractions in both groups. Positive correlation was not observed between t-25OHD and DBP, but it was noted between t-25OHD and albumin (Control: *r* = 0.33; hip fracture: *r* = 0.21).

### 3.2. Influences of Lifestyle and Major Chronic Diseases (MCD) on VD Levels

When lifestyle was taken into account, significantly (*p* < 0.001) higher 25OHD levels were found in the hip fracture group with active lifestyle (*N* = 101) than in the inactive group (*N* = 98), [t-25OHD: 37.3 (23.5–64.7) vs. 25.8 (12.6–45.1) nmol/L; b-25OHD: 3.3 (2.3–6.0) vs. 2.1 (1.0–4.1) nmol/L; f-25OHD: 8.9 (6.0–16.1) vs. 5.9 (3.1–1.3) pmol/L], respectively. 

Concentrations of three 25OHD fractions and related markers were investigated in the context of three main chronic diseases. Detailed results are given in electronic Supplementary Material 1 (Table S1). Here, only the significant differences are highlighted. The lowest t-25OHD levels were observed in patients with malignancies [29 (21–36) vs. 42 (37–46); *p* < 0.01] and in cases with chronic kidney disease [28 (21–35) vs. 41 (36–45); *p* < 0.05] when compared to patients without these diseases. Bioavailable and free 25OHD levels were lower in patients with malignant tumours than in patients without tumours. We did not observe notable differences of VD biomarker levels neither in patients with chronic liver disease nor in patients on oral anticoagulant therapy.

### 3.3. Relationship between the Investigated Markers and Functional Outcomes

**Figure 1:**
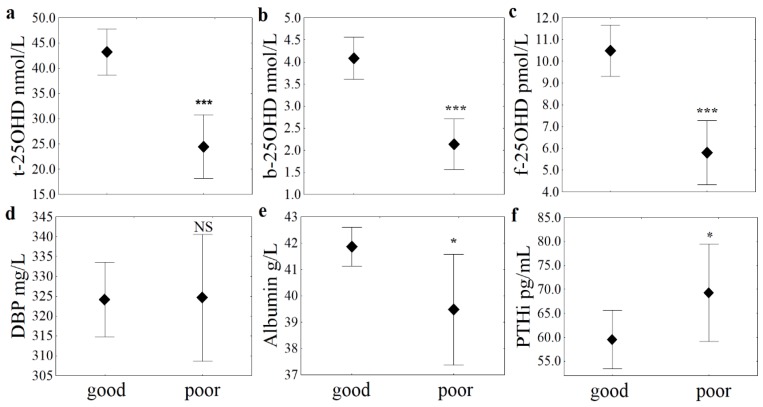
Levels of three 25OHD fractions and related markers in the context of post-surgery clinical condition measures. The levels of biomarkers are shown in mean ± 0.95 confidence interval. Abbreviations: NS: not significant (*p* = 0.914); the stars symbolize the level of significance (* *p* < 0.05: albumin *p* = 0.048; PTHi: *p* = 0.015; *** *p* < 0.0001) in the Mann-Whitney U Tests. (**a**) t-: total; (**b**) b-: bioavailable; (**c**) f-: free 25OHD vitamin; (**d**) DBP: vitamin D binding protein; (**e**) albumin; (**f**) PTHi: intact parathyroid hormone.

Patients with better functional performance scores after surgery (FPAS) showed significantly higher 25OHD levels of each fraction and higher albumin levels. In contrast, DBP levels did not show a notable relationship with the early clinical condition (Figure 1). While no difference in VD supply values were noted between sexes, worse FPAS measures were more frequent in men than in women (29% vs. 15%; *p* = 0.029).

Active lifestyle was the strongest inherent significant determinant factor (OR = 9.6_CI:3.8–24.1_; *p* < 0.001) for favorable FPAS during one-step regressions. Women have an almost three times higher chance (OR = 2.6_CI:1.3–5.2_; *p* = 0.007) for favorable early recovery than men. Age and major chronic diseases (MCD) showed not significant predictive property for FPAS.

We investigated the relations between ASA (American Society of Anesthesiologist) score and clinical condition, and also between ASA score and survival by one-step binary logistic regression, but didn’t find any significant association. 

All three 25OHD forms proved significant predictors for better performance related to inherent factors (gender, lifestyle), but lifestyle remained the strongest predictor (OR = 9.5–10.1) in all three models (Table 3). The chance of favorable early recovery was almost five times higher (OR = 4.6_CI:2.0–11.0_) and significantly (*p* < 0.001) when patients had higher than 4.5 pmol/L f-25OHD levels, controlled by significant inherent factors (Model-3). 

The odds ratio values for t-25OHD and b-25OHD levels were lower (3.7 and 2.9), but still significant (Model-1-2). Model-4 showed that patients with less than 73 pg/mL PTHi had a three times higher chance for better FPAS, than those who had higher PTHi levels (Table 3).

### 3.4. Results of Survival Analyses

We found four significant factors (FPAS, age, kidney and malignant diseases) for survival status using one-step, one by one-logistic regression. The chance to stay alive was more than five times higher for patients with good functional performance (OR = 5.7_CI:2.4–13.8_; *p* < 0.001) than for patients who had poor early recovery. Age inversely correlated with survival (OR = 0.95_CI:0.91–0.98_; *p* = 0.011). Patients without kidney or malignant diseases showed almost three times higher odds for survival (OR = 2.7_CI:1.2–6.0_; *p* < 0.012) than patients without these diseases. In contrast, liver disease and anticoagulant therapy did not influence survival.

Altogether associations of biochemical markers with survival time—controlled by functional performance, age and presence of kidney or malignant diseases—were not significant in the Cox regression analyses, certain trends were nevertheless noted (detailed results are not shown).

**Figure 2:**
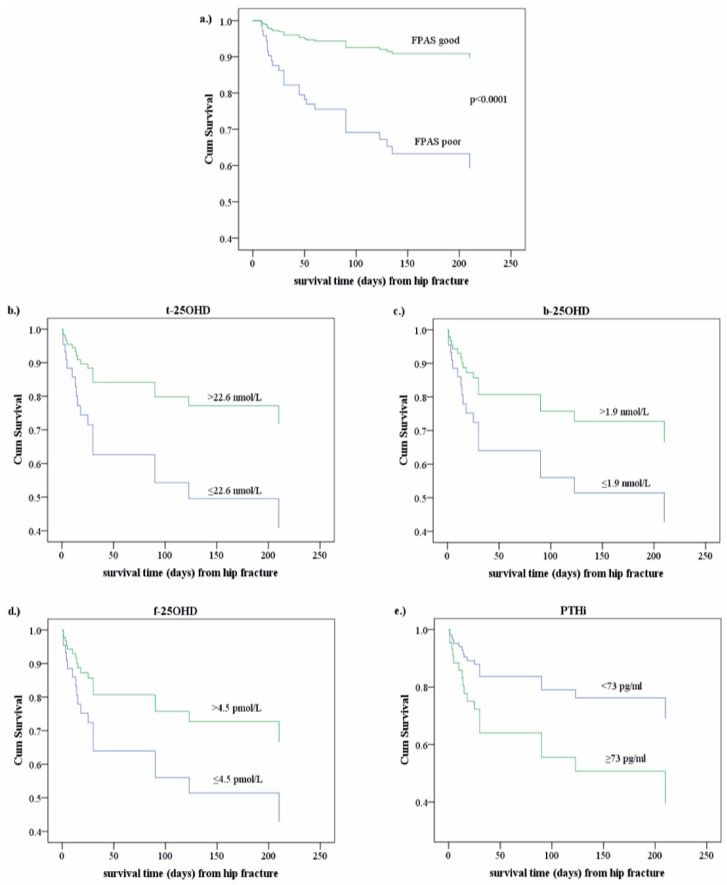
Effects on survival. (**a**) The effect of postoperative functional performance: Cum: cumulative, FPAS: functional performance after surgery. The chance to survive is more than six times higher (OR = 6.5_CI:3.2–13.3_; *p* < 0.0001) in patients with good FPAS than for patients with poor (FPAS), before discharged. Cox-regression was performed on adjusting age and kidney/malignant diseases; (**b**–**e**) Influence of favorable 25OHD fractions and PTHi levels on survival in patients with poor postoperative performance: (**b**) t-: total; (**c**) b-: bioavailable; (**d**) f-: free 25OHD vitamin; (**e**) PTHi: intact parathyroid hormone, Cum: cumulative; Cox regressions controlled by age and kidney and malignant diseases, concerning only 43 patients with poor functional performance after surgery. PTHi: *p* = 0.204; t-25OHD: *p* = 0.084; b-25OHD and f-25OHD: *p* = 0.178.

FPAS influenced survival so powerfully (Figure 2a), controlled by age and kidney disease/malignancy (OR: 6.5_CI:3.2–13.3_; *p* < 0.0001), that the relationship between biomarker levels and survival time appeared weak in the analyses. For this reason, we investigated the effects of biomarkers separately in 43 patients with poor FPAS using Cox regression and taking into consideration age, and kidney and malignant diseases. Figure 2b–e shows that higher 25OHD levels (b,c,d) and lower PTHi (e) support longer survival. 

**Figure 3:**
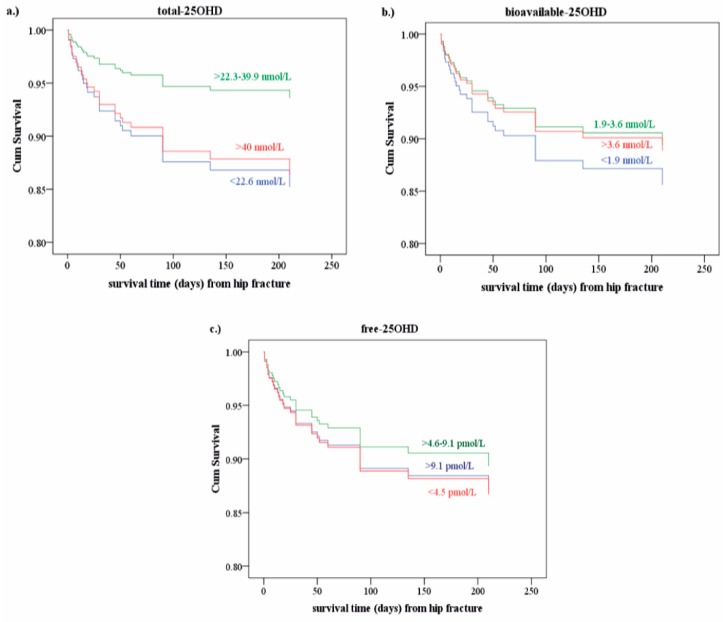
Ranges of circulating 25OHD forms and variability of cumulative survival times. (**a**) total-; (**b**) bioavailable-; (**c**) free-25OHD. The cumulative (cum) survival times depend on the 25OHD levels, especially of t-25OHD levels (trends without reaching statistical significance). The threshold values are given based on the values of our control group at 5th, 25th percentile.

The overall rate of mortality was 15.6%. When lifestyle was taken into account, the mortality rate decreased to 8.9% in patients with active lifestyle and increased to 22.4% in those with inactive lifestyle. The mortality rates differed along with increasing percentile levels of biomarkers, as determined based on control group values. With the increase of total and free 25OHD (from the values of less than 5th to 25th percentile), partially decreased mortality rates were observed, but from the values of 25th percentile the mortality rates increased again. A marginally negative relation was found between vitamin D binding proteins (DBP, albumin), b-25OHD levels and mortality: the lower the levels were, the higher the rate of mortality was. Figure 3 shows the cumulative survival rates depending on the 25OHD levels, most strikingly in case of the t-25OHD levels. However, all these findings appeared as trends only, without reaching statistical significance.

## 4. Discussion

In our aging society, one of the greatest public health problems is the treatment of frequent hip fractures and follow-up care for these elderly patients [2,4]. Many authors reported on the connection between fracture risk and VD deficiency [1,3,6,26]. Nevertheless, there is few data on the relation between VD status, postoperative healing [9,14,15,16,17] and survival [2,4,7,8]. All these studies focused on the t-25OHD levels without binding proteins and the outcome after surgery without considering lifestyle and MCD. Some authors emphasize that the validity of an overall 25OHD cut-off is limited [21,27,28]. Our hypothesis was that the common cut-off values might not be appropriate in every age group, especially in elderly patients suffering from different chronic diseases. That is why we analyzed various 25OHD fractions in patients with hip fracture in comparison with age-matched controls, and defined our own threshold limits that differed from the threshold values offered by the international guidelines. 

Our results demonstrate that deficient VD status rarely occurs in patients with hip fracture (34% vs. 72%) when the own control threshold value of t-25OHD is considered, instead of the guideline decision limit [25]. There are no significant differences among the three circulating 25OHD forms reflecting vitamin D supply, even correlated with PTHi values. The three 25OHD fractions have a significant effect on early recovery in different degrees. (OR: f-25OHD = 4.6; t-25OHD = 3.7; b-25OHD = 2.9) The free fraction seems to be a marginally better predictor for good early recovery than the other two. Di Monaco et al. [15] found an association between serum levels of t-25OHD, but not of f-25OHD levels, and stipulated functional recovery after hip fracture in 350 patients who had 17.3 nmol/L median t-25OHD levels. Wang et al. [17] reported similar results, but their threshold limit was higher (29.5 nmol/L) than ours (22.6 nmol/L). According to Liu et al. [14], 76 patients with an unfavorable functional outcome had lower serum t-25OHD levels, compared to those with better functional performance. 

We measured lower DBP and albumin levels in patients with hip fracture than in control persons. Some data suggest a significant connection between DBP values and the outcome of various diseases, which could be explained by the impacts of VD on immune functions [12]. Various studies have demonstrated a direct correlation between serum DBP levels and survival rates in polytraumatised patients as a part of the acute phase reaction [13,17,19]. 

Low albumin levels along with VD deficiency might be the consequence of unfavorable nutritional status or can occur because the tissues take up more albumin-bound 25OHD during acute phase reaction in patients. The positive correlation between albumin and t-25OHD levels prompted us to postulate that the binding proteins may control the regulation of 25OHD tissue uptake [1,22,28,29]. 

Our results also showed that the chance for good FPAS scores in women was almost three times higher than in men, in spite of the fact that women with hip fracture were older and their VD supply was the same as that of male patients. The gender differences in life expectancy are well known. The factors include biological as well as environmental factors; for example, differences of health behaviors [8,30], as recorded by several authors [2,9,14,16,31].

Lifestyle factors such as nutrition and physical activity have the greatest influence on bone health [4,16]. We confirmed that patients with active lifestyles had eight times higher chance of better recovery than patients with a passive mode of life. 

We performed comparative analyses of patients with and without MCD influencing VD metabolism: the lowest t-25OHD levels were observed in patients with malignancies and renal diseases. Levels of b-25OHD and f-25OHD were lower only in cases with malignant tumours. MCDs are independent factors for FPAS, but chronic kidney and malignant diseases significantly influence survival.

Our results unequivocally confirmed that the chance to survive is more than six times higher for patients with good FPAS than for patients who had poor early recovery after adjusting for possible confounders. Altogether, the associations of biochemical markers with survival times controlled by age and MCDs were observed as trends, but not as significant associations. Since FPAS influenced the survival most significantly, we also investigated the biomarkers separately in patients with poor early performance. We observed that lower PTHi and higher 25OHD levels are beneficial for survival, but approaching statistical significance only in case of t-25OHD.

The overall rate of mortality in this study was 15.6% comparable with literary data. When lifestyle was taken into account, the mortality rate decreased to 8.9% in our patients with active lifestyles and increased to 22.4% in patients with inactive life styles. Some authors found that 12–36% of patients with hip fracture died in the first year [4,7,8]. After this period, their mortality rates did not differ from those in the age-matched general population. In these observations, neither MCD nor lifestyle was taken into consideration [27]. Curiously, we observed better survival with levels of t-25OHD more than 22.6 nmol/L, but shorter survival times in patients with higher than 40 nmol/L levels. This fact calls attention to potentially different (lower and upper) cut-off values for VD supply in elderly people. For comparison, guideline recommendations suggest higher target values. We observed bad to worse survival rates associated with higher than 39.5 nmol/L of total, and higher than 9.2 pmol/L of free 25OHD levels, while with higher than 3.6 mmol/L of bioavailable 25OHD levels the survival rates were gradually getting better. According to some metaanalyses and Amrein et al. [32,33,34], who only investigated t-25OHD, it is possible to have a U-shaped relationship between the 90-day survival rates and the VD levels in the elderly [32]. Similar conclusions may be drawn from our results, although only for the lower ranges of the t-25OHD levels, but not for the b-25OHD levels. We postulate that the cause of this U-shaped relationship between VD levels and survival may be related to an altered role of albumin and tissue uptake of 25OHD in elderly people. Our observation of lower but also high levels of vitamin D associated with shorter survival call attention to a calibrated substitution of vitamin D. Pretreatment measurements of the 25OHD levels may guide intervention. It is also important to keep in mind that the circulating 25OHD levels depend on the season, lifestyle and eating habits of foods enriched with vitamin D. The clinician has to be informed about the consumption of these foods and also the patients’ outdoor habits. Another important point of consideration is that the need for vitamin D supplementation in elderly people is not only influenced by their age, but also by their various comorbidities.

## 5. Summary, Major Findings and limitations of Our Studies

Suboptimal VD status occurs twice more frequently in patients with hip fracture than in control individuals, considering our own control threshold value of 25OHD against values of international guidelines.

Measurements of bioavailable- and free-25OHD levels for the assessment of VD status are recommended only when chronic kidney and malignant diseases are present. 

Comorbidities involving chronic illnesses and aging should be taken into account for adequate VD substitution and optimal doses should be individually determined based on serum 25OHD levels. Because all 25OHD forms are independent predictors for FPAS, and survival is greatly influenced by FPAS, higher 25OHD levels are beneficial for survival. However, our observation adds that this favorable effect is true only in a given concentration range.

The rates of mortality decrease with increasing b-25OHD, DBP and albumin levels, while this tendency turns to the opposite in case of total and free 25OHD. 

Limitations of our studies include the following: (1) We did not estimate Bartel Index for the evaluation of the patients’ activities; (2) the 25OHD levels were not measured by a gold standard assay; nevertheless, the manufacturer of our assay declared that the measured values should correlate well with those obtained by mass spectrometry; (3) the main limitation is related to the cross-sectional study design. To determine whether or not our findings have any clinical utility, further studies are needed from well-designed trials to determine the effects of vitamin D for survival of patients with hip fracture in late-life. Finally, it should be noted that we were not able to use either logistic regression or Cox regression with more than three independent variables as inherent factors, because of the relatively low number of cases.

It would be more useful for clinical practice if surgical treatment and pre-surgical Hb levels could have been introduced into the multiple regression. Several excellent publications [35,36] draw attention to their significance.

## Figures and Tables

**Table 1:** Main characteristics of patients with hip fracture.

Registered Data	Female	Male	*p*	Total
	*N* = 132	*N* = 67		*N* = 199
	*N* (%)	*N* (%)		*N* (%)
Age (mean ± SD years)	77.7 ± 10.1	68.6 ± 11.9	<0.001 *	74.7 ± 11.5
Active lifestyle	67 (51)	36 (54)	0.692	103 (51)
Time of admission in sunny months (250–300 h/month)	108 (82)	57 (85)	0.564	165 (83)
Major chronic diseases (MCD) **:				
Liver disease	7 (5)	10 (15)	0.024	17 (9)
Kidney disease	21 (16)	5 (8)	0.07	26 (13)
Malignancy	27 (21)	15 (22)	0.443	42 (21)
without MCD	93 (71)	47 (70)	0.965	114 (57)
Other chronic diseases:				
DM	27 (21)	5 (8)	0.013	32 (16)
Hypertension	37 (28)	11 (16)	0.07	48 (24)
Cardiovascular disease	30 (23)	17 (25)	0.678	47 (24)
out of them on oral anticoagulant	19	11		30
Other diseases ***	27 (21)	20 (30)	0.14	47 (24)
Osteoporosis treated with bisphosphonates and calcium ****	44 (33)	6 (9)	<0.001	50 (25)
ASA physical status classification:				
ASA I	12 (9)	6 (10)	-	18 (9)
ASA II	80 (61)	42 (63)	-	122 (61)
ASA III	40 (30)	19 (29)		59 (30)
Fracture locations:				
Femoral neck	59 (45)	26 (39)	-	85 (43)
Trochanteric	73 (55)	41 (61)		114 (57)
Surgical treatment:				
Osteosynthesis	118 (89)	63 (94)	-	182 (91)
Prosthesis	14 (11)	4 (6)	-	18 (9)
FPAS:				
Good	111 (84)	45 (67)	0.029	156 (80)
Poor	21 (16)	22 (33)	0.006	43 (22)
Died during seven months follow up	17 (13)	14 (21)	0.141	31 (16)
Survival time median days (Q25–Q75)	30 (10–52)	15 (4–45)	0.449	19 (5–19)

Abbreviations: ** MCD: major chronic diseases that affect vitamin D metabolism; (Q25–Q75): the interquartile-range; FPAS: functional performance after surgery *** different neurological or psychiatric diseases, **** only 8 women were on vitamin D_3_ supplements. DM: diabetes mellitus; ASA: American Society of Anesthesiologist score; *p*: levels of significance by Khi2 or Fisher exact tests, * expect for age by Mann-Whitney U-test.

**Table 2:** Comparisons of laboratory markers in the groups with and without fracture.

Biomarkers	Hip Fracture *N* = 199	Control *N* = 102	*P*
t-25OHD nmol/L	32.5 (16.1–53.0)	58.2 (39.4–78.2)	<0.001
b-25OHD nmol/L	3.0 (1.5–5.0)	5.3 (3.6–7.8)	<0.001
f-25OHD pmol/L	8.0 (3.8–12.4)	13.1 (9.1–17.8)	<0.001
PTHi pg/mL	52.2 (38.8–76.5)		<0.001
	48.5 (37.0–69.0) *	42.3 (34.3–54.8)	0.004 *
DBP mg/L	324 ± 56	342 ± 58	0.009
Albumin g/L	41.4 ± 5.1	45.3 ± 3.9	<0.001
Ca mmol/L	2.31 ± 0.18	2.39 ± 0.11	<0.001
Ca-Alb	2.28 ± 0.18	2.28 ± 0.09	0.927

The results are given in mean ± SD and medians with interquartile-range (Q25–Q75); *p*-values were calculated by Mann-Whitney U test; * patients with chronic renal were failure excluded. *p*: levels of significance. Abbreviations: CaAlb: Calcium concentration corrected by albumin; t-: total, b-: bioavailable, f-: free 25OHD vitamin; DBP: vitamin D binding protein; PTHi: intact-parathyroid hormone.

**Table 3:** Relationship between post-surgery functional performance and biomarkers related to gender and lifestyle.

Independent Variables (Categorical Points of Reference)	Dependent Variable: Functional Performance after Surgery					
	B	SE	P	OR	95% CI for OR	
					Lower	Upper
**MODEL-1**						
**t-250HD** (≥22.6 nmol/L)	1.31	0.41	0.001	3.7	1.7	8.2
**Gender** (women)	1.32	0.42	0.002	3.8	1.6	8.6
**Lifestyle** (active)	2.29	0.51	<0.001	9.9	3.7	26.7
**MODEL-2**						
**b-250HD** (≥1.9 nmol/L)	1.07	0.41	0.009	2.9	1.3	6.4
**Gender** (women)	1.40	0.42	0.001	4.1	1.8	9.3
**Lifestyle** (active)	2.31	0.50	<0.001	10.1	3.8	27.0
**MODEL-3**						
**f-250HD** (≥4.5 pmol/L)	1.53	0.42	<0.001	4.6	2.0	10.5
**Gender** (women)	1.47	0.44	0.001	4.3	1.8	10.2
**Lifestyle** (active)	2.25	0.51	<0.001	9.5	3.5	25.7
**MODEL-4**						
**PTHi** (<73 pg/mL)	1.16	0.43	0.006	3.2	1.4	7.4
**Gender** (women)	1.36	0.42	0.001	3.9	1.7	8.9
**Lifestyle** (active)	2.47	0.50	0.000	11.9	4.5	31.5

The results of binary logistic regression analyses are presented. Abbreviations: B: coefficient of regression; SE: standard error; p: level of significance; OR (expected B): odds ratio; CI: confidence interval at 95%; t-: total, b-: bioavailable, f-: free 25OHD vitamin, PTHi: intact-parathyroid hormone.

## Supplementary Materials

The following are available online at http://www.mdpi.com/2077-0383/7/8/193/s1. Table S1: Results in relations to presence of major chronic diseases, which can influence vitamin D metabolism (*N* = 85).
