# Supplementary material for: Prognostic Relevance of Circulating 25OHD Fractions for Early Recovery and Survival in Patients with Hip Fracture
Source: J Clin Med. 2018 Aug 1;7(8):193. doi: 10.3390/jcm7080193 (PMC6111530; doi:10.3390/jcm7080193)
Supplement: Supplementary file 1 [file jcm-07-00193-s001.zip › jcm-329847-supplementary.docx]

ELECTRONIC SUPPLEMENTARY MATERIAL-1 (*Table S1*)

**Table S1**. Results in relations to presence of major chronic diseases, which can influence vitamin D metabolism (*N* = 85).

|  | **N** | **t-25OHD**  **nmol/L** | **b-25OHD**  **nmol/L** | **f-25OHD**  **pmol/L** | **PTHi**  **pg/mL** | **DBP**  **mg/L** | **Albumin**  **g/L** | **Ca-Alb**  **mmol/L** |
| --- | --- | --- | --- | --- | --- | --- | --- | --- |
| Chronic liver disease  yes  no  Chronic kidney disease  yes  no  Malignancy  yes  no | 17  26  42 | 41 (28-54)  39 (35-43)  28 (21-35)*  41 (36-45)  29 (21-36)**  42 (37-46) | 3.8 (2.5-5.2)  3.7 (3.3-4.1)  2.7 (1.8-3.5)^p^  3.8 (3.4-4.3)  2.8 (2.0-3.6)**  3.9 (3.5-4.4) | 10.0 (6.5-13.6)  9.5 (8.5-10.6)  7.4 (5.1-10)  9.9 (8.8-11)  7.3 (5.4-9.2)*  10.1 (9.0-11.3) | 56 (45-68)  64 (58-70)  105 (78-131)***  57 (52-62)  68 (53-83)  62 (56-68) | 315 (287-344)  325 (316-333)  317 (298-335)  325 (316-334)  314 (298-330)  327 (317-336) | 42 (40-44)  41 (41-42)  39 (37-42)*  42 (41-42)  41 (39-43)  42 (41-42) | 2.22 (2.12-2.32)  2.29 (2.26-2.31)  2.35 (2.25-2.46)  2.27 (2.25-2.30)  2.29 (2.21-2.37)  2.28 (2.26-2.30) |

The values are given as means (±95% Confidence limits of means). Abbreviations: *p*; almost significant (*p* = 0.06). (0.067–0.08). The levels of significance as calculated by the Mann-Whitney U test: * *p* < 0.05; ** *p* < 0.01; *** *p* < 0,001. Ca-Alb: Ca corrected by albumin.
